# Synaptic vesicles contain small ribonucleic acids (sRNAs) including transfer RNA fragments (trfRNA) and microRNAs (miRNA)

**DOI:** 10.1038/srep14918

**Published:** 2015-10-08

**Authors:** Huinan Li, Cheng Wu, Rodolfo Aramayo, Matthew S. Sachs, Mark L. Harlow

**Affiliations:** 1 Department of Biology, Texas A&M University, TAMU 3258, College Station, TX 77843-3474 USA.

## Abstract

Synaptic vesicles (SVs) are neuronal presynaptic organelles that load and release neurotransmitter at chemical synapses. In addition to classic neurotransmitters, we have found that synaptic vesicles isolated from the electric organ of *Torpedo californica,* a model cholinergic synapse, contain small ribonucleic acids (sRNAs), primarily the 5′ ends of transfer RNAs (tRNAs) termed tRNA fragments (trfRNAs). To test the evolutionary conservation of SV sRNAs we examined isolated SVs from the mouse central nervous system (CNS). We found abundant levels of sRNAs in mouse SVs, including trfRNAs and micro RNAs (miRNAs) known to be involved in transcriptional and translational regulation. This discovery suggests that, in addition to inducing changes in local dendritic excitability through the release of neurotransmitters, SVs may, through the release of specific trfRNAs and miRNAs, directly regulate local protein synthesis. We believe these findings have broad implications for the study of chemical synaptic transmission.

Multiple downstream events occur upon the activity-dependent release of neurotransmitter at chemical synapses. Most obviously, the presynaptic release of neurotransmitter leads to a stereotypic electrical change across a postsynaptic cell membrane. Thus at vertebrate neuromuscular junctions the release of acetylcholine leads to the activation of nicotinic acetylcholine receptors on the muscle membrane, membrane depolarization and subsequent muscle contraction^1^,^2^,^3^. More dynamically, the presynaptic release of neurotransmitter coupled with coincident local postsynaptic membrane depolarization leads to a change in synaptic physiology that can persist for minutes, hours or days^4^. These long term changes have been best characterized at central nervous system (CNS) synapses, and can lead to long term potentiation (LTP) or depression (LTD) of the synaptic coupling between the two cells. In the short-term (minutes) both LTP and LTD rely upon changes in calcium, but for these synaptic changes to be consolidated for the long-term (hours and days) requires, in addition to calcium influx, local protein synthesis^5^.

Local protein synthesis at the synapse requires a host of mRNAs, translation factors, and ribosomes^6^,^7^,^8^,^9^. In addition, it is suspected that microRNA (miRNA) and other non-coding RNA (ncRNA) that include, but are not restricted to, endogenous small interfering RNA (esiRNA), piwi-interacting RNA (piRNA), antisense and long-ncRNA, play a key role in regulating translation^10^. Mechanistically, the release of neurotransmitter presynaptically has been thought to indirectly drive the selective control of postsynaptic protein synthesis through activity-based modulation of calcium^11^.

We hypothesized that the presynaptic terminal might play a more direct role in the regulation of postsynaptic transcription and translation. Previous studies have identified sRNAs that are associated with synaptosomes, as well as sRNAs that are released from and taken into synaptosomes and sRNAs that associate with SV fractions^12^,^13^. As a first step to test the hypothesis that the presynaptic terminal might play a more active role in local protein synthesis, we looked for the presence of, and ultimately sequenced, small molecule RNAs (sRNAs) that not only associate with synaptosomes and SVs, but localize within the SVs. We first chose SVs isolated from the electroplaques of *Torpedo californica*, a classic peripheral nervous system (PNS) preparation that provides an abundance of cholinergic synaptic vesicles from one class of motor neuron^14^. We treated the SVs with RNase to remove any exogenous cytoplasmic RNA from the preparation, and after RNA extraction, found an abundance of sRNAs protected from degradation. Sequencing of the sRNAs derived from affinity purified SVs revealed the presence of 5′ tRNA fragments, with a 5′-fragment of glutamyl-tRNA (tRNA^Glu^) sequence constituting the most abundant of the sequences. We verified by northern blot that the 5′ fragments were not a result of the RNase treatment of the vesicles, and that the fragments were protected from degradation in the absence of (but not the presence of) detergent. *In situ* hybridization of the most abundant fragment sequence confirmed the presence of the fragment in the axons and presynaptic terminals of the electroplaque. We extended the results to SVs isolated from the mouse CNS. As with the electroplaque, we found an abundance of sRNA species that were co-enriched with SVs and were resistant to RNase degradation. The 5′-fragment of tRNA^Glu^ that was most abundant in cholinergic *T. californica* SVs was the second most abundant species of sRNA found in SVs isolated from the mouse brain. Other species of sRNAs were found to be abundant in mouse CNS vesicles, including known miRNAs, and most abundantly, 5′ RNA fragments of the Ro ribonucleoprotein associated Y1 RNA (RNY1)^15^. Together these observations not only support the idea that sRNAs are present within SVs, they also suggest that these sRNAs play key roles regulating local protein synthesis at the synapse.

## Results

### Cholinergic vesicles isolated from the electric organ contain RNA

**Figure 1:**
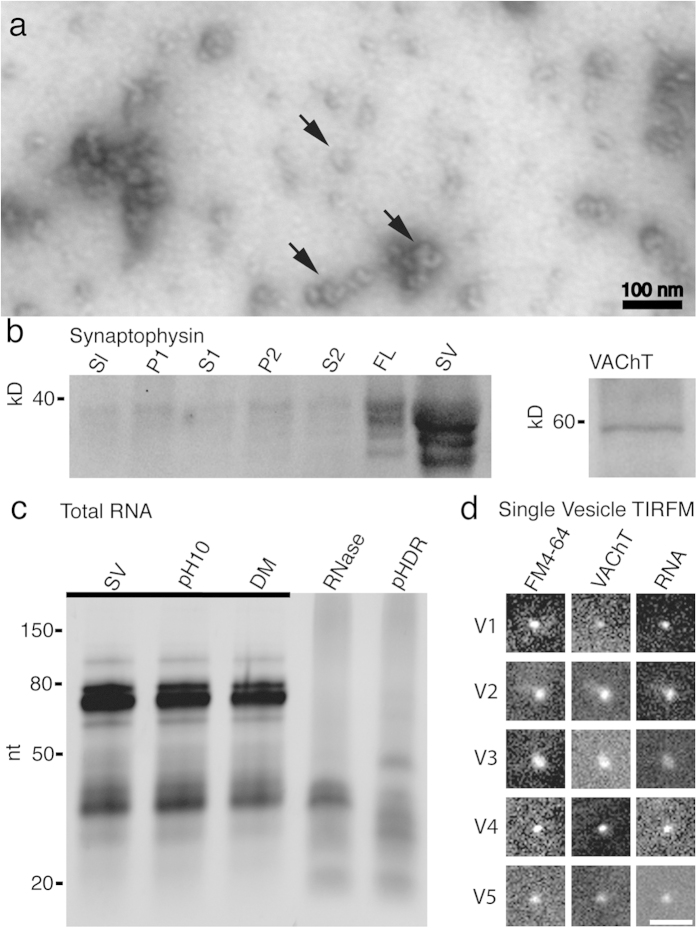
Cholinergic SVs from the electric organ of *Torpedo californica* contain RNA. (**a**) Electron micrograph of negatively stained SVs isolated and enriched from electroplaque tissue. Sample contains abundant ~80 nm vesicles (some marked with arrows). (**b**) Western-blot analysis of the synaptic vesicles during purification. The SV protein synaptophysin was used as a marker during the enrichment. The isolation procedure includes the collection of the original slurry (Sl), two centrifugation supernatants and pellets (S1, P1 and S2, P2), followed by a sucrose density gradient centrifugation and collection of the SV fluffy layer (FL). Further purification using size-exclusion chromatography yields the final, enriched sample of SVs (SV). Purified vesicles were tested by immunoblot and found to be positive for the ~60 kD vesicle acetylcholine transporter (VAChT). (**c**) Abundant sRNAs co-enrich with the synaptic vesicles (SV). These sRNAs are stable under high pH (pH10), or in the presence of detergent (DM). After addition of RNase much of the RNA is degraded; however an RNase resistant ~32 nt band persists (RNase). The RNase resistant band of RNA can be degraded in the presence of high pH, detergent, and RNase (pHDR). Bands of gel underlined (_) indicate a 20-fold reduction of sample loaded. (**d**) TIRF microscope images of SVs reveals that single SVs contain RNA, as demonstrated by triple-labeling of the SVs with the styryl dye FM4–64, VAChT, and the RNA dye SYTO12. Five representative vesicles shown from a total of 307. Scale bar = 1 μm.

We isolated synaptic vesicles from the electric organ of the Pacific ray *T. californica* in order to provide an abundant, homogenous preparation of cholinergic SVs^14^. We chose a freeze grinding method of isolation that has been shown by others to retain more of the SV neurotransmitter content while offering a similar SV enrichment (~20 fold) as other isolation procedures^16^,^17^. In addition, we wanted to isolate SVs residing within classic synaptosomal boutons as well as those present at less structured synaptic varicosities. SVs were collected from the middle of the 0.6 M (1.07 g/ml density) sucrose gradient layer, well above the 1.2 M (1.17 g/ml) sucrose layer used to isolate exosomes^18^,^19^ or detect exosome markers^20^,^21^. The size of the vesicles we isolated averaged ~80 nm (Fig. 1a), larger than SVs within the vertebrate CNS (~40 nm)^22^ or SVs found at vertebrate neuromuscular junctions (~50 nm)^23^,^24^, but normal for vesicles from this preparation^25^. As further verification that the isolated vesicles were neuronal in origin, we found by western blot analysis that the synaptic vesicle marker synaptophysin^26^ was enriched during isolation and that the vesicular acetylcholine transporter (VAChT)^27^ was present in the final preparation (Fig. 1b; Supplemental Fig. 1).

We hypothesized that, in addition to neurotransmitter, SVs might contain RNA. To test this hypothesis we extracted RNA from the SV enriched preparation using a TRIzol extraction method and found abundant small molecule RNAs (Fig. 1c). The cytoplasm of presynaptic terminals likely contains RNA molecules that might co-enrich with the SVs. In order to test whether the sRNAs were exogenous to or resided within the SVs we hypothesized that the vesicle membranes would provide protection from RNase degradation, thus allowing us to separate the cytoplasmic associated RNA from any vesicular protected RNA. We tested the preparation under five conditions. In the presence of high pH buffer (pH 10—known to disrupt RNA secondary and tertiary structure^28^) we observed no difference between the RNA isolated from the pH 7.4 SV preparations and the RNA isolated from the pH 10 SV preparation. We next chose, along with high pH, to add membrane detergent (DM; n-Decyl-β-D-Maltopyranoside) to disrupt the SV membranes without denaturing proteins^29^. Once again in the presence of pH 10 and DM we found no difference between the RNA we isolated from the untreated SV preparation. Next we added RNase to the SVs. In the presence of RNase we found a substantial (~20 fold; Supplemental Table 1) reduction in RNA, suggesting that exogenous RNA does co-enrich with SVs. After RNase treatment a specific band of sRNAs of approximately ~32 nucleotide (nt) persisted. It was only when we combined pH 10, DM, and RNAse that we observed degradation of the ~32 nt band (Fig.1c).

We have previously used a single-vesicle imaging approach to study the co-localization of multiple types of vesicular transporters to single vesicles in the cholinergic SV preparation^30^,^31^. As an initial step to verify that the sRNAs not degraded by RNase resided within cholinergic synaptic vesicles, and were not the result of RNA co-enriching with the SVs during the isolation procedure, we triple labeled isolated SVs in the presence of RNase (Fig. 1d). Isolated SVs were labeled by immunofluorescence with a polyclonal antibody against VAChT, the nucleic acid dye SYTO12, and the membrane steryl dye FM4–64. Because SVs are smaller than the diffraction limit, cholinergic vesicles were identified as small diffraction-limited dots visible in the FM4–64 channel that co-labeled for VAChT. Almost all cholinergic SVs co-labeled for RNA (96.1%; N = 307).

### Cholinergic vesicles isolated from the electric organ contain tRNA fragments

**Figure 2:**
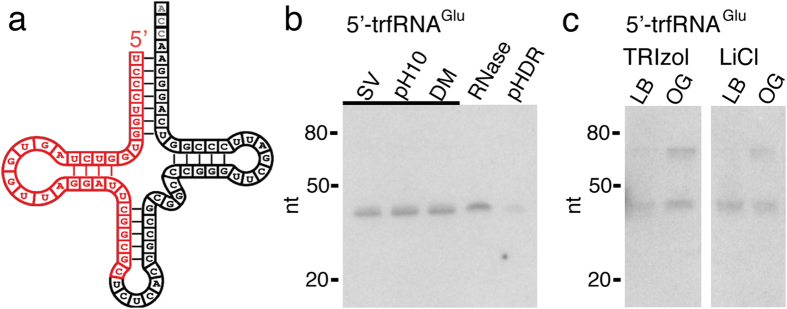
Cholinergic vesicles isolated from the electric organ of *T. californica* contain 5′-tRNA fragments. (**a**) Next-generation sequencing of RNA isolated from RNase treated SVs enriched and affinity purified against VAChT reveals that cholinergic SVs contain 5′ fragments of tRNA. The most abundant fragment (5′-trfRNA^Glu^) shown in red mapped onto the complete tRNA glutamate (tRNA^Glu^_CUC_) shown in black. (**b**) Northern analysis of RNA isolated from SVs verifies that the 5′-trfRNA^Glu^ sequence was not a product of RNase treatment. RNA isolated from SVs purely isolated (SV), at pH10 (pH10), in detergent (DM), treated with RNase (RNase), and treated simultaneously with pH10, detergent, and RNase (pHDR). Bands of gel underlined (_) indicate a 20-fold reduction of sample loaded. (**c**) Northern analysis of total RNA (TRIzol) and tRNA enriched RNA (LiCl) isolated from the electric lobe (LB) and electric organ (OG) of *T. californica* reveals full length tRNA^Glu^_CUC_ and 5′-trfRNA^Glu^ were found in near equal abundance in the electric organ, whereas 5′-trfRNA^Glu^ was more abundant than tRNA^Glu^_CUC_ in the electric lobe.

We sequenced the RNase resistant cholinergic SV’s sRNA. As a further step beyond size-and density-based purification methods, magnetic beads with conjugated antibodies against VAChT were used in the purification procedure (Supplemental Fig. 1)^19^,^32^. SV non-luminal RNA was removed by treatment with RNase before luminal RNA was extracted by TRIzol. Five primary sequences with slight variations of length dominated the next generation sequencing reads, accounting for 60% of the total reads (Table 1). The most abundant primary sequence was that of a 5′ fragment of a tRNA^Glu^ with the anticodon CUC (tRNA^Glu^_CUC_) that is conserved across vertebrates (Fig. 2a), followed by 5′ fragments of two different tRNAs specifying glycine (tRNA^Gly^_CCC_), a 5′ fragment of tRNA Valine (5′-tRNA^Val^_CAC_), and a 5′ fragment of tRNA Lysine (tRNA^Lys^_UUU_). Fragments of tRNA, both 5′ and 3′, are signaling molecules cleaved by the vertebrate RNase angiogenin^33^,^34^,^35^, and are designated 5′- and 3′- trfRNAs (for stress-induced tRNA fragment). We verified that the 5′-trfRNAs were not a result of the RNase treatment used in the isolation procedure by northern analysis of the most abundant 5′-trfRNA^Glu^ sequence (Fig. 2b; Supplemental Fig. 2). The northerns were conducted three times, each with RNA isolated from SVs prepared from separate fish. Only a ~32 nt band was observed in samples of the isolated SVs, pH 10 treated SVs, membrane detergent (DM) treated SVs, and RNase treated SVs. RNAs from the SV sample treated with pH 10-DM-RNase were degraded, providing further evidence that the 5′-trfRNAs reside within the cholinergic SVs, and are not a product of the RNAse treatment (Fig. 2b).

To test whether the full-length tRNA^Glu^_CUC_ could be found in the presynaptic electric lobe and/or the postsynaptic electric organ we extracted total RNA from the two tissues for northern anaylsis. Full-length tRNA^Glu^_CUC_ and 5′-trfRNA^Glu^ were found in near equal abundance in the electric organ, whereas 5′-trfRNA^Glu^ was more abundant than tRNA^Glu^_CUC_ in the electric lobe (Fig. 2c; Supplemental Fig. 2). We further enriched for tRNA from total RNA preparations using sodium acetate and LiCl extraction^36^, and observed a similar pattern of tRNA^Glu^_CUC_ and 5′-trfRNA^Glu^ abundance in the tissues (Fig. 2c; Supplemental Fig. 2).

### 5′-trfRNA^Glu^ localizes to axons and presynaptic nerve terminals

**Figure 3:**
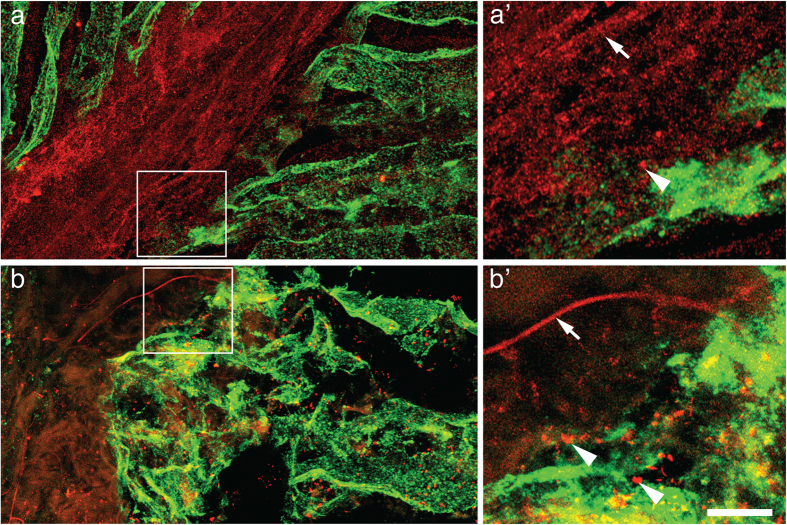
5′-trfRNA^Glu^ localize to axons and presynaptic nerve terminals of the electric organ of *T. californica.* (**a**) Immunohistochemical labeling of 20-micron thick cryostat sections of the electric organ. Axons and presynaptic terminals labeled with polyclonal antibodies against VAChT (red). The nicotinic acetylcholine receptor rich surface of the electrocyte cells labeled with α-Bungarotoxin (green). (**a’**) Enlarged region from (a), axon marked with white arrow and presynaptic bouton white arrowhead (**b**) A similar pattern of labeling is present within *in situ* hybridization samples of the same tissue. Axons and presynaptic boutons labeled with a probe for 5′-trfRNA^GLU^ (red), and the surface of the electrocyte cells labeled with α-Bungarotoxin (green). (**b’**) Enlarged region from (b), axon marked with white arrow and presynaptic boutons white arrowheads (**a’**,**b’**) scale bar = 5 μm.

To confirm that 5′-trfRNAs are presynaptic SV-associated species, we performed fluorescent *in situ* hybridization on cryostat sections of the *T. californica* electric organ. Probes for 5′-trfRNA^Glu^ or the SV transporter VAChT were used in association with fluorescently labeled α-Bungarotoxin, a postsynaptic marker for the nicotinic receptors that cover the surface of the electric organ. Nerve axons run perpendicular to the pancake stacked electric organ electrocytes before turning 90 degrees and innervating the entire surface of each cell. We found that the probe for 5′-trfRNA^Glu^, but not a scrambled control probe, labeled the axons and surface of the electrocytes in a similar pattern as the presynaptic SV marker VAChT (Fig. 3a,b; Supplemental Fig. 3), suggesting that the 5′-trfRNA^Glu^ is associated with axons and presynaptic nerve terminals rather than the postsynaptic electric organ.

### Synaptic vesicles isolated from mouse CNS contain RNA

**Figure 4:**
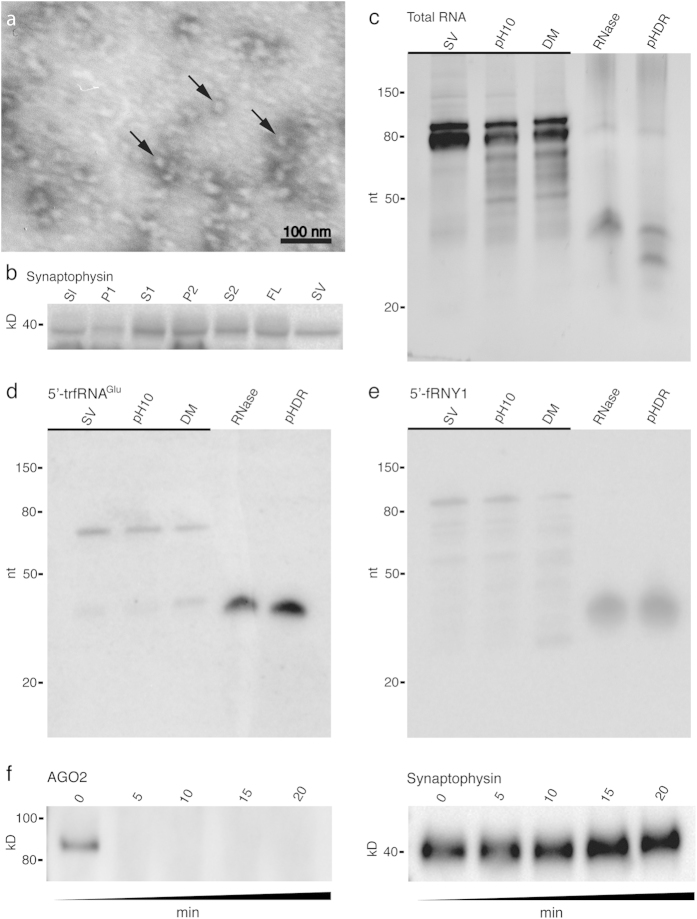
SVs isolated from the mouse CNS contain sRNAs. (**a**) Electron micrograph image of negatively stained vesicles isolated from the mouse CNS. Sample contains abundant ~40 nm vesicles (some marked with arrows). (**b**) Western-blot analysis of the synaptic vesicles during purification. The SV protein synaptophysin was used as a marker during isolation. The isolation procedure includes the collection of the orginal slurry (Sl), two centrifugation supernatants and pellets (S1, P1 and S2, P2), followed by a sucrose density gradient centrifugation and collection of the SV fluffy layer (FL). Further purification using size exclusion chromatography yields the final, isolated sample of SVs (SV). (**c**) Abundant sRNAs co-enrich with the synaptic vesicles (SV). These sRNAs are stable under high pH (pH10), or in the presence of detergent (DM). After addition of RNase much of the RNA is degraded; however an RNase resistant ~32 nt and lower ~20 nt band persists (RNase). The RNAse resistant band of RNA can be partially degraded in the presence of high pH, detergent, and RNAse (pHDR). (**d**) Northern analysis of RNA isolated from SVs verifies that the 5′-trfRNA^Glu^ sequence, the second most abundant sequence in mouse CNS SV, was not a product of RNase treatment. RNA isolated from SVs purely isolated (SV), at pH10 (pH10), in detergent (DM), treated with RNase (RNase), and treated simultaneously with pH10, detergent, and RNase (pHDR). In the mouse CNS, both full-length tRNA^Glu^_CUC_ and 5′-trfRNA^Glu^ co-enrich with the SVs, but only 5′-trfRNA^Glu^ is resistant to RNase treatment. (**e**) The most abundant species of sRNA in mouse was a 5′ fragment of a Y RNA: Ro-associated Y1 (RNY1):5′-fRNY1. Like the 5′-trfRNA^Glu^, full length and precursor sequences of RNY1 co-enrich with the SVs. (**c**–**e**) Bands of gel underlined (_) indicate a 20-fold reduction of sample loaded. (**f**) The miRNA and RISC associated protein AGO2 co-enriches with SVs, however it is susceptible to trypsin degradation (0–20 minute exposure) indicating it does not reside within the lumen of the SVs. The SV protein synaptophysin, which has trypsin cleavage domains residing within the lumen of the SVs, is not degraded.

We hypothesized that synaptic vesicles residing in the CNS might also contain sRNAs. To test this, we isolated SVs from the mouse brain of Swiss Webster mice. As expected, the mouse brain SVs were ~40 nm in diameter (Fig. 4a) and the synaptic vesicle marker synaptophysin co-purified with SVs during isolation (Fig. 4b; Supplemental Fig. 4). Once again, SVs were collected from the middle of the 0.6 M (1.07 g/ml density) sucrose gradient layer, well above the 1.2 M (1.17 g/ml) sucrose layer used to isolate exosomes^18^,^19^ or detect exosome markers^20^. We treated the mouse SVs with TRIzol and found that the preparation was enriched in sRNAs (Fig. 4c). As with the *T. californica* SVs, we tested the mouse SV RNA under three conditions. We raised the buffer to pH10, but saw little difference. Next we added the detergent DM (also at pH10) and observed little difference between the RNA we isolated from the SV prep. When we added RNase to the SV prep we had a dramatic reduction (~38 fold; Supplemental Table 2) in the amount of total RNA extracted; however, two bands of resistant RNA at ~32 nt and ~21 nt remained. These bands appear to be partially degraded when we combined pH10, membrane detergent (DM), and RNase (Fig. 4c).

We sequenced the RNase resistant mouse SV sRNA using next generation sequencing. Two classes of sequences with slight variations of length dominated the reads, with the top ten sequences accounting for 40% of the total reads (Table 2). The most abundant set of sequences mapped to a 5′ fragment of a Y RNA: Ro-associated Y1 (RNY1)^15^. The 5′ fragment of RNY1 (5′-fRNY1) is part of the stem loop of RNY1, and like the 5′-trfRNAs, exists as multiple copies of similar length (~32 nt). The second most abundant set of sRNAs in the mouse CNS SVs was the most abundant sequence found in the *T. californica* SVs: the 5′-trfRNA^Glu^ (~32 nt). The third, fourth, seventh, eighth and ninth mapped to neuronal associated microRNAs, including miR128, miR99, miR100, miR22, and miR127 (21–22 nt). Fragments of other tRNA^Glu^ species accounted for the fifth and sixth most abundant sequences. The fifth sequence does not map perfectly to any known mouse genomic sequence for a tRNA, perhaps as a result of a splice variation or mouse genomic strain difference, and the sixth most abundant sequence mapped to a fragment of tRNA^Glu^_TTC_. The tenth most abundant sequence mapped to the 5′-terminal fragment of the 28 s ribosomal RNA (20 nt). These nucleotides, along with the last 20 nt of the 5.8 s RNA, form the eukaryotic expansion segment known as ES4^L^, a double-stranded complex of the 60 s ribosomal subunit^37^.

We verified that the 5′-trfRNAs were not a result of the RNase treatment used in the isolation procedure by northern analysis with the 5′-trfRNA^Glu^ sequence. The northerns were conducted three times, each with RNA isolated from a different preparation of mouse SVs. As with the *T. californica*, the 32 nt 5′-trfRNA^Glu^ is present in the mouse SV preparations before RNase treatment (Fig. 4d). Unlike the *T. californica* SV preparation, full-length ~80 nt tRNA^Glu^_CUC_ does appear to associate with synaptic vesicles prior to RNase degradation. In addition, we did not see an appreciable decline in signal for the 5′-trfRNA^Glu^ after pH 10-DM-RNase treatment (Fig. 4d; Supplemental Fig. 5). We can quantify the number of 5′-trfRNA^Glu^ per SV from the mouse CNS using the weight of an average mouse SV (25.6 × 10^−18^ g/vesicle)^38^ and the amount of 5′-trfRNA^Glu^ detected in our northern analysis. We found that on average, 15 vesicles per 1000 would contain a 5′-trfRNA^Glu^ if the sRNAs were evenly distributed (Supplemental Fig. 5). In the northern analyses of the 5′-fRNY1 RNA, multiple sizes representing full length and partial fragments of the RNY1 are associated with the SVs before RNAse treatment—ranging from shorter than 30 nt to the full length 112 nt (Fig. 4e; Supplemental Fig. 6). However, only the 32 nt 5′-fRNY1 is substantially enriched inside the SV and thus protected from RNase degradation. We found that on average, 142 vesicles per 1000 would contain a 5′-fRNY1 if the sRNAs were evenly distributed (Supplemental Fig. 6). As with the 5′-trfRNA^Glu^, we did not see an appreciable degradation of the 5′-fRNY1 after pH 10-DM-RNase treatment. We verified that the RNAse cocktail was capable of degrading all of the sRNA in the sample by first isolating the RNase resistant RNA with Trizol followed by a treatment of RNase. Once separated from the SV, all of the sRNAs were degraded (Supplemental Fig. 7).

We did not find any complementary sequences to the 20–21 nt miRNAs found within the SVs, suggesting the miRNAs present are single stranded. We tested whether the miRNAs that reside within the SVs might be associated with argonaute-2 (AGO2), as a previous study found AGO2 associated with enriched SVs^13^. AGO2 bound to miRNAs can form the minimal RNA-induced silencing complex (RISC)^39^. We found that AGO2 was associated with our SV preparation. To test whether AGO2 resided within the SVs, or was associated with the exterior of the SVs, we treated the sample with the protease trypsin. We hypothesized that much like the RNase resistant sRNAs, AGO2 would be protected from degradation if it resided within the SVs. AGO2 was not protected from degradation, suggesting the miRNAs present are not associated with AGO2 (Fig. 4f). To verify that we were not inducing the degradation of the vesicles with the application of trypsin we also tested for the degradation of synaptophysin. Synaptophysin can be cleaved by trypsin, but the trypsin cleavage sites reside within the lumen of the SVs. Synaptophysin was not degraded in the trypsin treated samples (Fig. 4f). That AGO2 is associated with the isolated SVs raised the possibility that some of the RNase and detergent resistant sRNAs might be in an AGO2 associated complex. To test that hypothesis we treated SVs with trypsin for 20 minutes, followed by trpsin inhibitor and RNase. The sRNAs were resistant to the trypsin treatment (Supplemental Fig. 7). As an additional test we treated the SVs with trypsin in association with the membrane detergent DM for 20 minutes before addition of trypsin inhibitor and RNase treatment. The RNase resistant sRNAs persisted - northerns for both 5′-fRNY1 and 5′-trfRNA^Glu^ demonstrated that the sRNAs were not affected by prior treatment with trypsin, and the addition of DM had no effect (Supplemental Fig. 7).

### What is the structure of 5′-trfRNA?

**Figure 5:**
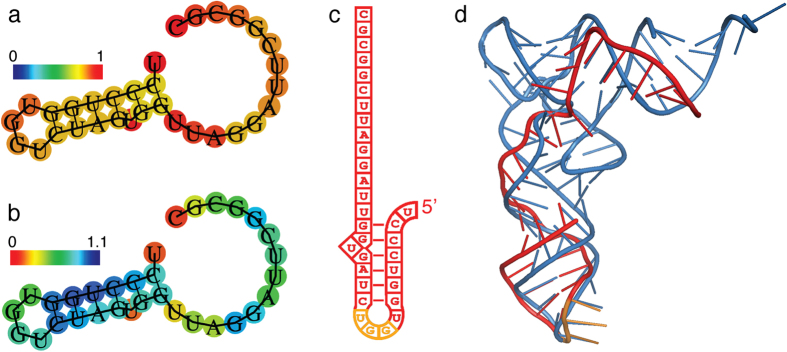
Tertiary structure of 5′-trfRNA^Glu^ mimics tRNA anticodon stem and loop. (**a**) RNAfold was used to calculate the minimum free energy secondary structure of 5′-trfRNA^Glu^. Base-pair probabilities are shown in a heat map from 0–1; for non-paired nucleotides, heat map indicates probability of being unpaired. (**b**) The centroid secondary structure is identical to the minimum free energy structure. The heat map shows the positional entropy (from 0–1.1). (**c**) Secondary structure of 5′-trfRNA^Glu^ showing the pseudo-anticodon region in orange. (**d**) Tertiary structure of 5′-trfRNA^Glu^ calculated using RNACOMPOSER (red and orange). The tertiary structure was aligned in pymol to a known crystal structure of a tRNA, shown in blue (yeast tRNA^Phe^ 1ehz) The stem and loop structure determined in this manner closely resembled known crystal structures of the tRNA anticodon stem and loop, particularly at the anticodon region (orange). The sequence of this pseudo-anticodon is GGU. Both RNAfold and RNACOMPOSER used with default settings.

In an effort to gain insight into the molecular mechanism of 5′-trfRNA^Glu^, we examined the predicted structure of 5′-trfRNA^Glu^ through computational approaches. We used RNAfold^40^ to calculate the optimal secondary structure of the minimum free energy (MFE) of the sequence (Fig. 5a). We found that MFE of the optimized secondary structure was −5.20 kcal/mol. The free energy of the thermodynamic ensemble was −5.37 kcal/mol, with a frequency of the MFE structure in the ensemble of 76% (Fig. 5a). A similar centroid secondary structure was calculated with a MFE of −5.20 kcal/mol. Based upon this secondary structure we calculated a tertiary structure of the 5′-trfRNA^Glu^ using RNACOMPOSER^41^. Surprisingly, the stem and loop structure determined in this manner closely resembled known crystal structures of the tRNA anticodon stem and loop, particularly at the anticodon region (Fig. 5d), even though the tRNA^Glu^ anticodon from the full-length tRNA is not present in the 5′ tRNA fragment. The sequence of this pseudo-anticodon is GGU and thus would recognize threonine codons, a codon not present in vertebrate genomes.

## Discussion

In this study, we demonstrated that cholinergic SVs isolated from the PNS electroplaque of *T. californica* contain sRNAs (Fig. 1c). Single vesicle imaging of RNase treated SV preparations from *T. californica* demonstrated that membrane bound structures co-labeled with probes for membrane, for cholinergic neurotransmitter transporter VAChT, and for RNA (Fig. 1d). We immunopurified *T. californica* SVs using antibodies against VAChT. We then isolated RNase resistant sRNAs from these vesicles. Next generation sequencing showed that these sRNAs consisted primarily of 5′-tRNA fragments, with 5′- trfRNA^Glu^ being the most abundant (Table 1). We demonstrated by northern analysis that the 5′- trfRNA^Glu^ was not a byproduct of RNase treatment (Fig. 2b), and that it exists as both a full-length tRNA^Glu^_CUC_ and a 5′-trfRNA^Glu^ in the *T. californica* PNS, and in the electric lobe, the location within the CNS of the cell bodies of the electromotor neurons (Fig. 2c). In addition, we demonstrated by northern analysis that the signal representing 5′- trfRNA^Glu^ in the *T. californica* SV preparation could only be reduced below detectable levels in the presence of detergent, high pH, and RNase (Fig. 2b), suggesting that this RNA resides within the vesicles*. In situ* hybridization of the 5′- trfRNA^Glu^ sequence was consistent with immunohistochemical labeling of the SV marker VAChT in cryostat sections of the *T. californica* electric organ (Fig. 3a,b).

We extended our findings to heterogeneous SVs isolated from the mouse brain. We found that these mouse brain SVs also contain sRNAs, the most abundant of which is a 5′ fragment of the RO associated Y1 RNA, we term 5′-fRNY1 (Table 2). The second most abundant sRNA sequence in SVs isolated from the mouse brain is the 5′- trfRNA^Glu^. We demonstrated by northern analysis that the 5′-fRNY1 and the 5′- trfRNA^Glu^ were not a byproduct of RNase treatment (Fig. 4d,e), i.e. we found that precursors for both fragments exist, but are degraded by RNase treatment, suggesting they do not reside within the SV lumens. One potential interpretation for the presence of the precursors in association with the SVs is that the presynaptic terminal is a site of cytoplasmic maturation and cleavage of SV sRNAs. Finally, we demonstrated by northern analysis that the signals representing the 5′-fRNY1 and 5′- trfRNA^Glu^ in the mouse CNS SV preparation were RNase resistant (Fig. 4d,e)—even if the SVs were treated with trypsin (Supplemental Fig. 7). We do not have an explanation for the persistence of the mouse sRNAs in detergent and RNase compared to the *T. californica* sRNAs sensitivity to the same treatment. The smaller size of the mouse SVs may make them less soluble in detergent, or the sRNAs are in a trypsin resistant complex in the mouse SVs.

The most abundant sRNA present in *T. californica* SVs, as well as a large percentage of the sRNAs present in mouse SVs (second, fifth and sixth most abundant), were previously identified as stress-induced tRNA fragments (Tables 1 and 2). Two pathways are known to produce these fragments in vertebrates. 5′-trfRNA^Tyr^ arises by induced RNA pol III transcription in response to H_2_O_2_^42^. A second pathway for the production of 5′-trfRNAs is by the stress-induced RNase angiogenin^33^,^34^,^43^. Angiogenin has been shown to be involved in the fragmentation of tRNA^Glu^_CUC_ into 5′-trfRNA^Glu^ in airway epithelial cells after viral infection and induce stress granule formation and suppress protein translation in a miRNA and siRNA independent manner^35^. Whether a similar mechanism occurs at the synapse, or in other tissues shown to be enriched in 5′-trfRNA^Glu^ and 5′-trfRNA^Gly^ is not known^44^. We calculated a tertiary structure of the 5′-trfRNA^Glu^ using RNACOMPOSER^41^, which predicted a stem and loop structure that closely resembled known crystal structures of the tRNA anticodon stem and loop (Fig. 5d). If 5′-trfRNA^Glu^ does alter translation in a miRNA and small interfering RNA (siRNA) independent manner, perhaps it does so by directly interacting with the protein translation machinery by mimicking the anti-codon stem loop of a tRNA.

In this paper we focused on the isolation and localization of sRNAs isolated from SVs of two different organisms, the PNS of *T. californica* and the CNS of mouse. The most abundant sequences of sRNAs isolated and sequenced were over 30 nt; however, we did isolate and sequence miRNAs in the 20–21 nt range, including miR128, miR99a, miR100, miR22, and miR127. The most abundant miRNA, and the third most abundant class of sequences was miR128, a miRNA important for neuronal development, synaptogenesis, and post-mitotic neuronal functioning^45^. Members of the miR-99 family (miR99a, and miR100, the fourth and seventh most abundant mouse sRNAs) are miRNAs that have been shown to co-enrich with polyribosomes in mammalian neurons, and regulate the mammalian target of rapamycin (mTOR) pathway^46^. miR22, the eighth most abundant mouse sRNA, is important for cerebellar development, and in adults has been shown to protect neurons from neurodegeneration, and is down regulated in both Huntington’s and Alzheimer’s disease^47^. miR127, along with a cluster of miRNAs found on chromosome14q32, is maternally expressed, and the down regulation of miRNAs within this cluster (including miR127) has been linked to schizophrenia^48^. The mechanism by which miRNAs are taken in at the synapse is not known. However, a previous study has demonstrated that miR99a is released from synaptosomes in an activity and calcium dependent manner, consistent with the release from SVs, and synthetic miRNAs were taken up by synaptosomes via an unspecified endocytic pathway^13^.

Long-term changes in synaptic plasticity require protein synthesis, the local dendritic regulation of which is still an active area of research^6^,^7^,^8^,^9^. One common, and critical component of every model of local protein synthesis is the role activity plays in the up-regulation and down-regulation of translation. Activity at a local synapse is driven by the presynaptic fusion of synaptic vesicles with the plasma membrane, and the subsequent release of the contents residing within the SV lumen. Currently most of our understanding of SV content release has focused on small molecule neurotransmitters that rapidly bind metabotropic and ionotropic receptors leading to near instantaneous changes in target membrane excitability. Given the immense specificity and wide ranging effects that sRNAs can exert on transcription and translation^10^,^49^,^50^, the precise molecular control with which SVs link presynaptic activity to SV content release, and the developmental precision with which the pre and postsynaptic apparatus are spatially aligned at every type of synapse, the addition of sRNAs to the list of molecules released by SVs during exocytosis, and/or possibly taken up by SVs during endocytosis, is both powerful and exciting. It provides the potential for a direct means of controlling translation locally, which may prove at least as important as the proposed calcium induced mechanisms for controlling translation.

## Materials and Methods

### Isolation and enrichment of synaptic vesicles

Methods were adapted from Ohsawa^51^. Three preparations of electric organ with tissue from 3 separate *Torpedo californica* fish (2 female, 1 male), and three preparations of mouse brains (approximately 50 mouse brains of both male and female per each preparation) were used during these studies. A Spex Freezer Mill 6800 (Spex Sample Prep; Metuchen, NJ) was cooled to −180 °C and ~25 g of frozen electric organ from an individual (Aquatic Research Consultants; San Pedro, CA) or 25 g frozen Swiss Webster mouse brains (BioChemed Services, Winchester, V.A.) was ground with 25 g of frozen buffer pellets (320 mM Sucrose, 10 mM TRIS-Cl, pH 7.4) (Sigma-Aldrich; St. Louis, MO). The resulting powder of buffer and electric organ/brain was warmed to 4 °C with 50 ml of buffer solution (320 mM Sucrose, 10 mM Tris-Cl, pH 7.4, 4 °C). The resulting slurry (100 ml) was centrifuged at 20,000 rpm for 10 minutes (Beckman Coulter JA-20 rotor—Avanti J25 centrifuge) (Beckman Coulter; Brea, CA). The resulting supernatant was centrifuged at 34,000 rpm for 40 minutes (70ti rotor - Optima X80; Beckman). The supernatant was then loaded onto a 4 ml/4 ml 0.6 M/1.2 M sucrose step gradient (10 mM Tris-Cl, pH 7.4), then centrifuged at 48,000 rpm for 2 hours (70ti rotor - Optima X80). The 4 ml 0.6 M (1.07 g/ml density) sucrose fluffy layer, enriched in vesicles, was collected. Heavier densities and pellet (>0.6 M sucrose), known to be enriched in exosomes, were discarded^18^,^20^. A 2 ml sample of enriched vesicles was filtered using a 0.22 μm spin column (Spin-x, Corning; Corning, NY) to remove any large debris. The filtrate was injected into a Pharmacia LC500 plus FPLC (GE Healthcare, Fairfield, CT) and run through a 25 cm 4% agarose bead column (Bioscience Beads; West Warwick, RI). Separate bead columns were prepared for electric organ and mouse brains to ensure no contamination. The FPLC was eluted with a buffer solution PBS, pH 7.4 at a flow rate of 1.0 ml/min. The second major peak was collected, and the vesicles concentrated to a protein concentration of 5 mg/mL (measured by Bradford Assay)(Bio-Rad Laboratories, Inc.; Hercules, CA) using a Stirred Cell apparatus with a 100 kDa filter (PLHK02510; Millipore, Billerica, MS).

### Electron microscopy

Negative stain of isolated synaptic vesicles of *T. californica* and mouse are conducted following Jahn and Maycox^22^ . Briefly, A 5 μl sample of enriched synaptic vesicles (further concentrated to 20 mg/ml with Pierce concentrator, PES, 100 K MWCO (88503, Thermo Scientific) as determined by Bradford assay was pipetted onto a slot grid. Excess sample was removed by filter paper, and the grid was briefly washed in 12 mM sodium phosphate buffer, followed by 2 s staining with uranyl acetate (Ted Pella; Redding, CA), followed by another wash^22^. The slot grid was viewed with a JEOL 1200 JEOL Ltd., Akishima-Shi, TKY Japan) operated at 100 kV. Image was collected at 50,000 × magnification on a bottom-mounted 3072 × 3072, slow scan, lens-coupled CCD camera SIA 15C (SIA; Dulith, GA).

### Western blots

Synaptic vesicle lysates were loaded on to a 4–20% SDS-PAGE gel (456–9034; BioRad) and blotted using standard protocols. All gel lanes were loaded with equal volume (3 μl) of sample. Primary antibodies against synaptophysin (1:1000; MAB5258; EMD Millipore, Billerica, MA), AGO2 (1:1000; ab186733; Abcam, Cambridge, England), VAChT (1:1000; ab68986; Abcam), 58 K (1:1000; ab19072; Abcam), and Calnexin (1:1000; ab22595; Abcam). Secondary antibodies were HRP-conjugated goat-anti rabbit (1:2000; 2–348; EMD Millipore), rabbit anti-goat (1:2000; 6020–05, Southern Biotech; Birmingham, AL), and goat-anti mouse (1:2000; ab5879; Abcam). Visualization of blots was conducted with ECL Prime (RPN2232; GE Healthcare) according to manufacturer’s instructions. Chemiluminescence was quantified using a Chemicdoc XRS + Imager with Imagelab software (BioRad).

### Synaptic vesicle RNA isolation and tRNA isolation

SVs were isolated and concentrated as described above. For each assay, 1 preparation of vesicles was utilized and split into 5 equal samples. Each sample consisted of 3 × 50 μl of SVs (5 mg/ml) in PBS (80 mM Na_2_HPO_4_ and 25 mM NaH_2_PO_4_, 100 mM NaCl pH 7.4; Sigma). The 5 parallel treatments conducted on the SV preparations were: 1) addition of 50 μl PBS, pH 7.4, 2) addition of 50 μl PBS pH 11 (adjusted with NaOH; Sigma), 3) addition of 50 μl PBS pH 11 and 100 mM membrane detergent DM (n-Decyl-β-D-Maltopyranoside, D322; Anatrace, Maumee, OH), 4) addition of 50 μl PBS pH 7.4 with 1 μl RNase cocktail as instructed (RNase A (500 U/ml) and RNase T1 (20,000 U/ml), AM2286; Ambion/Life Technologies), and finally 5) addition of 50 μl combined pH 11, DM, and RNAse treatments. RNA was extracted after the treatments using 900 μl TRIzol (Invitrogen/Life Technologies) followed by 200 μl chloroform and 400 μl isopropanol (EMD Millipore), with a final precipitation by 75% ethanol. The total SV RNA was resuspended in 6 μl of DI water, and the three samples of each treatment were quantified—Supplemental Tables show combined results.

RNA concentration was determined using QuantiFluor RNA System (E3310, Promega, Madison, WI) and Quantus Fluorometer (E6150, Promega). Briefly, 1 μl of isolated RNA is dissolved for 5 minutes in 199 μl of Master solution (20X TE Buffer, DI water, and QuantiFluor RNA dye); the solution was then read in the Quantus Fluorometer.

To isolate total RNA from the electric lobe (CNS) and electric organ (PNS) of *T. californica*, 100 mg of tissue of each organ was dissected and ground with silica beads before RNA extraction by TRIzol (described above). For further enrichment of sRNA and tRNA, samples of total RNA were resuspended in 300 μl of 10 mM sodium acetate (Sigma-Aldrich) pH 4.5 at 4 °C, and mixed with 30 μl of 8 M LiCl (Sigma-Aldrich). After centrifugation at 15,000 g, 4 °C, the tRNA and sRNA is collected from the resulting supernatant.

### Fluorescent immunohistochemical labeling of isolated synaptic vesicles and TIRF imaging of single vesicles

Isolated SVs were passed through a 0.22 μm spin column, and 100 μl of synaptic vesicles (1 mg/ml) were transferred into 300 μl PBS (pH 7.4), and labeled with one round of primary and secondary antibody following the procedure^52^. Briefly, the vesicles were incubated for overnight with 1 μg of VAChT antibody in 4 °C, incubated for 30 min with 20 μl anti-rabbit IgG beads (Sigma-Aldrich), briefly centrifuged, and the vesicle containing supernatant was then incubated with 0.5 μg goat anti-rabbit secondary antibody labeled with Pacific Blue (P-10994, Life Technologies) for 4 hours before finally being incubated with 20 μl anti-goat IgG beads (Sigma-Aldrich). The beads were pelleted and the supernatant collected for imaging. SVs were incubated in RNase cocktail for 30 minutes.

FM4–64^53^ and SYTO 12 (S7574; Life Technologies) were added to the labeled vesicles (final concentration 1 μM), and the samples were settled on a glass bottom culture dish (MatTek P35G-1.5–20-C; Ashland, MA) for at least 1 hour. Settled vesicles were imaged with a Zeiss Axio Observer Z1 Microscope with TIRF slider, 100X TIRF objective (NA 1.45). Images were acquired using AxioVision (Carl Zeiss; Oberkochen, Germany). Three separate images from each field were taken using laser lines and filter cubes paired to eliminate fluorescent cross talk between the dyes: laser line 401 with filter cube 73 HE was used for Pacific Blue, laser line 488 with filter cube 38 HE was used for SYTO 12, and laser line 561 with filter cube 74 HE was used for FM 4–64. Images were collected with a Roper S/W PVCAM EMCCD camera and analyzed using ImageJ (NIH, Bethesda, MA) software. Suitable spots detected in the FM4–64 channel were marked. The other channels were then quantified for label.

### RNA gels and northern blots

Electrophoresis and blotting are previously described^36^. Denatured RNA samples were run through 10% TBE Urea precast gel (BioRad, 456–6033). Because of resulting sample differences in yield between non-RNAse treated and RNAse treated SV RNA preparations, gel lanes loaded with non-RNase treated samples were reduced by ~20-fold to match the amount loaded in the RNase treated lanes as described in figure legends and documented in Supplemental Materials. Northern blots were conducted in triplicate, in order to reduce bias from any single preparation, each northern was conducted on RNA isolated from a single fish. For mouse brain SVs, three separate preparations of SV RNAs were isolated in order to perform northerns in triplicate. Northern blots were transferred to Zeta Probe Blotting Membrane (BioRad, 162–0153) in 1xTBE buffer at 20 V for 90 min. The membrane was rinsed with 5xSSC buffer and RNAs were crosslinked to the membrane using a Stratalink UV crosslinker (auto setting). The membrane was incubated in a hybridization tube with 15 ml of prehybridization buffer at 42 °C for 3 or more hours. 50 pmol of oligo was radioactively labeled using 300 μCi [gamma32-P] ATP (NEG502A; Perkin Elmer; Waltham, MA) and T4 Polynucleotide Kinase and the signal was detected by autoradiography. Denatured probe (heated at 95 °C for 5 min and chilled on ice) was added to the hybridization tube, and incubated at 42 °C overnight. The membrane was washed with 10 ml 2xSSC/0.1% SDS and then with 100 ml 0.2xSSC/0.1% SDS at room temperature for 5 min, respectively. The membrane was washed at 42 °C with 100 ml 0.2xSSC/0.1% SDS for 30 min and then at 68 °C with 100 ml 0/1xSSC/0.1% SDS for 30 min. The membrane was dried and then exposed to a storage phosphorplate (GE Healthcare) for 16 h before being scanned with a Typhoon Trio Variable Mode Imager (GE Healthcare) and quantified based upon binding of probe to positive control loaded sample of known quantity using ImageQuant TL software (GE Healthcare).

### 20x SSC buffer: 3 M sodium chloride, 300 mM sodium citrate

Prehybridization buffer: 0.75 M NaCl, 50 mM NaPO4, 5 mM EDTA, 1%SDS, 50% formamide, 0.2% BSA, 0.2% polyvinylpyrollidone 40, 0.2% Ficoll 400 (Sigma)

trfRNA^Glu^—probe 5′- CGCCGAATCCTAACCACTAGCCACCA

trfRNA^Glu^—positive control *in vitro* transcription

5′-TAATACGACTCACTATAGGTCCCTGGTGGTCTAGTGGTTAGGATTCGGCGC

5′-GCGCCGAATCCTAACCACTAGACCACCAGGGACCTATAGTGAGTCGTATTA

Ro Y1—probe 5′ -AACTCACTACCTTCGGACCAGCC

Ro Y1—positive control *in vitro* transcription

5′-TAATACGACTCACTATAGGGGCTGGTCCGAAGGTAGTGAGTT

5′- AACTCACTACCTTCGGACCAGCCCCTATAGTGAGTCGTATTA

### RNA isolation and sequencing

SVs from *T. californica* and mouse whole brain were isolated and treated with RNAse cocktail (Ambion/Life Technologies) as described above. As a further enrichment, SVs from *T. californica* were affinity enriched using dynabeads (100.07D; Dynabeads; Invitrogen/ Life Technologies) with VAChT antibody^19^,^32^. The total RNA was extracted following the TRIzol procedure described above. Library preparation and sequencing were performed by the Genomic Sequencing and Analysis Facility at the University of Texas, Austin. The samples were prepared for sequencing using the TruSeq small RNA sample preparation kit (Illumina; San Diego, CA). Single-end reads (100 bp) were sequenced on an Illumina HiSeq 2500(Illumina).

### trfRNA *in situ* hybridization

For *in situ* hybridization (ISH), frozen *T. californica* electoplaque was cryosectioned (30–40 μm) and mounted onto Superfrost Plus slides (VWR, Radnor, PA). Fluorescein-labeled anti-sense morpholino oligomer probe matching coding 5′-trfRNA^GLU^ was synthesized from Gene Tools (Philomath, OR). Control fluorescein-labeled anti-sense cRNA probes matching coding 5′-trfRNA^GLU^ and scrambled were synthesized from IDT (Coralville, IA). Probes were hybridized to sections as previously described^54^, with minor modifications in amplification strategy. Following overnight hybridization, slides were incubated with alkaline phosphatase conjugated anti-fluorescein antibody (1:5000; 11426338910; Roche Life Sciences, Indianapolis, IN) for overnight at 4 °C. Tissues were washed and incubated in Fast Red (11496549001; Roche Life Sciences) according to manufacturer’s instructions for overnight at 4 °C in dark. Confocal images were captured with a Zeiss 780 Structure Illumination microscope (Zeiss, Germany). Sequences used for probe generation are listed below.

trfRNA^Glu^ Morpholino: 5′- GCCGAATCCTAACCACTAGACCACC-fluoroscein

trfRNA^Glu^ DNA control: 5′- GCCGAATCCTAACCACTAGACCACC-fluoroscein

trfRNA^Glu^ DNA scramble: 5′ - CCCGAATCGTAACGACTAGAGCAGC-fluoroscein

### Trypsin sensitivity

AGO2/synaptophysin Westerns: SVs were isolated and concentrated as described above. One preparation of vesicles was split into 10 equal samples. Each sample consisted of 5 μl of SVs (6 mg/ml) in PBS, pH 7.4. Paired samples were treating in the following manner: no Trypsin treatment, 2 μl Trypsin (T1426, 10 mg/ml; Sigma-Aldrich) treated for 5 mins, 10 mins, 20 mins or 30 mins. Westerns were conducted using antibodies against synaptophysin or AGO2.

SV RNA sensitivity to Trypsin: SVs were isolated and concentrated as described above. For each assay, 1 preparation of vesicles was utilized, and split into 4 equal samples of 50 μl. Each sample consisted of SVs (6 mg/ml) in PBS (80 mM Na_2_HPO_4_ and 25 mM NaH_2_PO_4_, 100 mM NaCl), pH 7.4. The 4 parallel treatments conducted on the SV preparations were: 1) addition of 50 μl PBS, pH 7.4, 2) addition of 50 μl PBS pH 7.4 and 0.5 μl Trypsin (T1426,10 mg/ml; Sigma-Aldrich) for 20 minutes, 3) addition of 50 μl PBS pH 7.4 and 0.5 μl Trypsin for 20 min, followed by 5 μl Trypsin Inhibitor (T9128, 6.5 mg/10 ml, Sigma-Aldrich) and 1 μl RNAse for 30 min., 4) addition of 50 μl combined Trypsin, 200 mM DM for 20 min, followed by 5 μl Trypsin Inhibitor and RNAse treatment for 30 minutes. RNA was extracted after the treatments and quantified as described above.

### Prediction of 5′-trfRNA^Glu^ structure

The secondary structure of the 5′-trfRNA^GLU^ was predicted using the default settings of RNAfold^40^ (ViennaRNA Package 2.0; Theoretical Biochemistry Group, University of Vienna Wien, Austria). The resulting secondary structure was used to calculate a predicted tertiary structure using the default settings for RNACOMPOSER^41^ (Institute of Computing Science, Poznan University of Technology, Poznan Poland). Visualization of the structure was conducted using the MacPymol Molecular Graphics System, Version 1.7.4 (Schrödinger, LLC; Cambridge, MA) running on a Mac Pro with OS 10.9.5 (Apple; Cupertino, CA).

## Additional Information

**How to cite this article**: Li, H. *et al.* Synaptic vesicles contain small ribonucleic acids (sRNAs) including transfer RNA fragments (trfRNA) and microRNAs (miRNA). *Sci. Rep.* **5**, 14918; doi: 10.1038/srep14918 (2015).

## Supplementary Material

Supplementary Information

## Figures and Tables

**Table 1:** sRNAs identified through next-generation sequencing of affinity purified SVs from the electroplaque of *T. californica.*

	Sequence	NT	Copy#	Identity
1	UCCCUGGUGGUCUAGUGGUUAGGAUUCGGCGC	32	2988724	5′-trfRNA^Glu^
	UCCCUGGUGGUCUAGUGGUUAGGAUUCGGCGCUC	34	475743	
	UCCCUGGUGGUCUAGUGGUUAGGAUUCGGCGCU	33	402173	
	UCCCUGGUGGUCUAGUGGUUAGGAUUCGGCG	31	249273	
	UCCCUGGUGGUCUAGUGGUUAGGAUUCGGC	30	80234	
			4196147	
2	GCAUUGGUGGUUCAGUGGUAGAAUUCUCGCC	31	829169	5′-trfRNA^Gly^
	GCAUUGGUGGUUCAGUGGUAGAAUUCUCGCCU	32	412080	
	GCAUUGGUGGUUCAGUGGUAGAAUUCUCGCCUG	33	299964	
	GCAUUGGUGGUUCAGUGGUAGAAUUCUCGCCUGC	34	205669	
	GCAUUGGUGGUUCAGUGGUAGAAUUCUCGC	30	172506	
			1919388	
3	GCAUCGGUGGUUCAGUGGUAGAAUUCUCGCC	31	200366	5′-trfRNA^Gly^
	GCAUCGGUGGUUCAGUGGUAGAAUUCUCGCCUGC	34	115460	
	GCAUCGGUGGUUCAGUGGUAGAAUUCUCGCCUG	33	79351	
			395177	
4	GUUUCCGUAGUGUAGUGGUUAUCACGUUCGCC	32	219845	5′-trfRNA^Val^
5	GCCCGGAUAGCUCAGUCGGUAGAGCAUCAGAC	32	130889	5′-trfRNA^Lys^

**Table 2:** sRNAs identified through next-generation sequencing of SVs isolated from the mouse CNS.

	Sequence	NT	Copy#	Identity
1	GGCUGGUCCGAAGGUAGUGAGUUAUCUCAAUU	32	4491149	RNA, Ro-associated Y1
	GGCUGGUCCGAAGGUAGUGAGUUAUCUCAAU	31	3921692	
	GGCUGGUCCGAAGGUAGUGAGUU	23	799788	
	GGCUGGUCCGAAGGUAGUGAGUUAUCUCA	29	347449	
	GGCUGGUCCGAAGGUAGUGAGUUAUCUCAA	30	338822	
	GGCUGGUCCGAAGGUAGUG	19	314918	
			10213818	
2	UCCCUGGUGGUCUAGUGGUUAGGAUUCGGCG	31	2289788	5′-trfRNA^Glu^
	UCCCUGGUGGUCUAGUGGUUAGGAUUCGGCGC	32	2039321	
	UCCCUGGUGGUCUAGUGGUUAGGAUUCGGC	30	882725	
	UCCCUGGUGGUCUAGUGGUUAGGAUU	26	576141	
	UCCCUGGUGGUCUAGUGGUUAGGAUUCGG	29	333204	
			6121179	
3	UCACAGUGAACCGGUCUCUUU	21	2581783	MIR-128-1
	UCACAGUGAACCGGUCUCUUUU	22	1857626	
	UCACAGUGAACCGGUCUCUUUUU	23	427156	
			4866565	
4	AACCCGUAGAUCCGAUCUUGU	21	2055063	MIR-99a
	AACCCGUAGAUCCGAUCUUGUG	22	346662	
			2401725	
5	UCCCUGUGGUCUAGUGGUUAGGAUUCGGCG	30	1488662	5′-trfRNA^Glu^
6	UCCCACAUGGUCUAGCGGUUAGGAUUCCUGGUU	33	1087820	5′-trfNA^Glu^
7	AACCCGUAGAUCCGAACUUGUG	22	545334	MIR-100
	AACCCGUAGAUCCGAACUUGU	21	350473	
			895807	
8	AAGCUGCCAGUUGAAGAACUGU	22	716387	MIR-22
9	UCGGAUCCGUCUGAGCUUGG	20	687125	MIR-127
10	CGCGACCUCAGAUCAGACGU	20	609557	rRNA-RNA, 28S ribosomal 5
