# Supplementary material for: Synaptic vesicles contain small ribonucleic acids (sRNAs) including transfer RNA fragments (trfRNA) and microRNAs (miRNA)
Source: Sci Rep. 2015 Oct 8;5:14918. doi: 10.1038/srep14918 (PMC4597359; doi:10.1038/srep14918)
Supplement: Supplementary Information [file srep14918-s1.pdf]

Synaptic vesicles contain small ribonucleic acids (sRNAs) including transfer RNA fragments (trfRNA) and microRNAs (miRNA)

Authors: Huinan Li, Cheng Wu, Rodolfo Aramayo, Matthew S. Sachs, and Mark L. Harlow

Department of Biology
Texas A&M University
TAMU 3258
College Station, TX 77843-3474

Corresponding Author:
Mark L. Harlow, Ph.D.
Department of Biology
Texas A&M University
TAMU 3474
College Station, TX 77843-3474
Email: mharlow@tamu.edu

Number of Pages: 12
Number of Figures: 7
Number of Tables: 2
Number of Supplemental Tables and Figures: 9

Conflict of Interest: The authors declare no competing financial interests

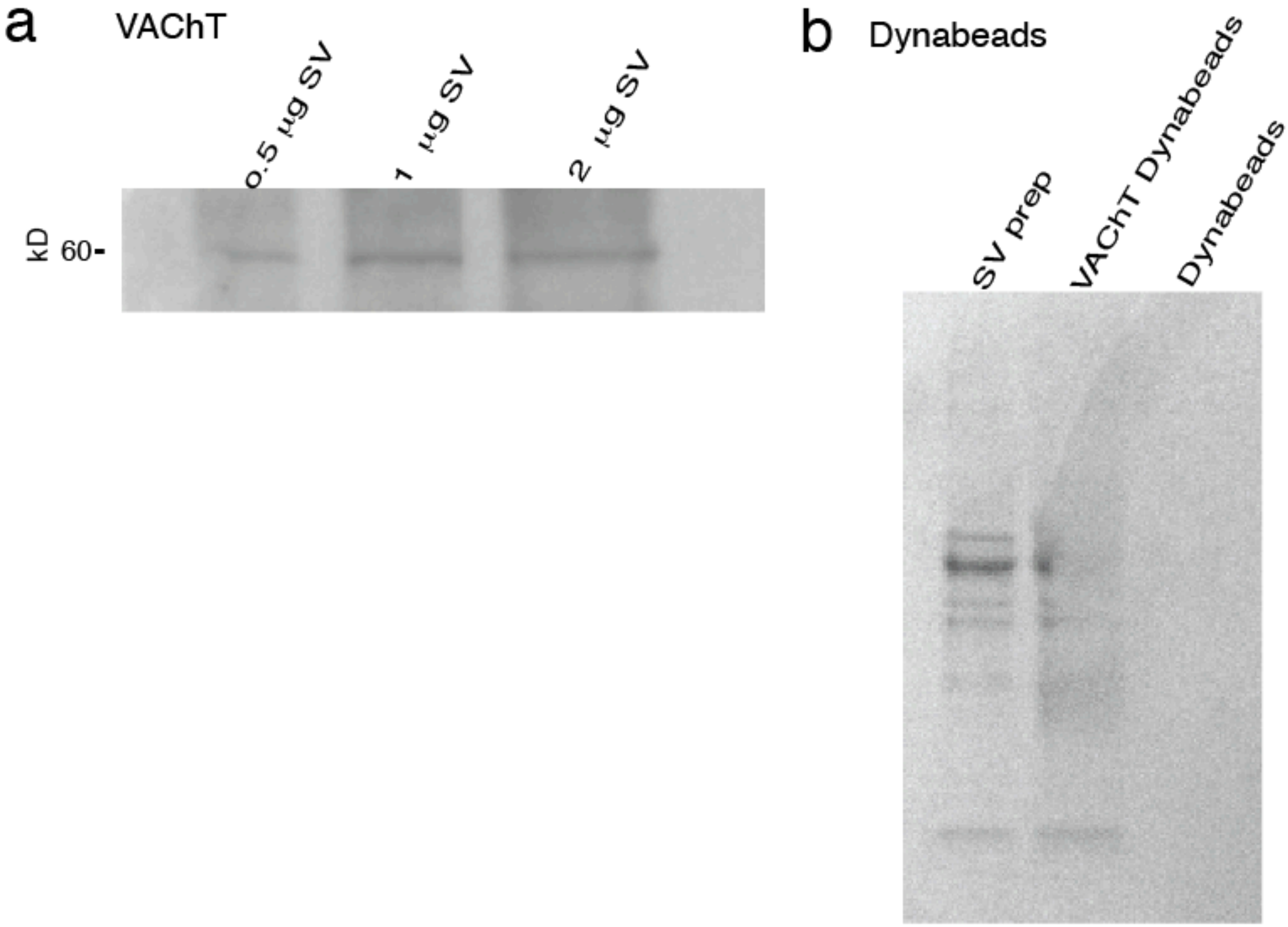

Supplemental Figure 1: Western blot analysis of purified synaptic vesicles from the electric organ of *Torpedo californica* and Ponceau staining of synaptic vesicles isolated with Dynabeads. a) The ~60 kd Vesicular AcetylCholine Transporter (VAChT) is shown in three lanes. Lane 1) 0.5 µg SV loaded 2) 1 µg SV loaded and 3) 2 µg of SV loaded. Further verification of antibody with this preparation described in Li & Harlow, 2014[31]. b) A protein gel containing three lanes of samples were transferred to a membrane and total protein was imaged with Panceau stain (G-Biosciences; St Louis, MO). SV lane shows 5 µg of purified synaptic vesicles, VAChT Dynabeads lane shows resulting affinity purified synaptic vesicles isolated with VAChT conjugated dynabeads, and Dynabeads lane shows parallel experiment demonstrating dynabeads without antibody conjugated do not isolate SVs or other material.

Supplemental Table 1: Quantification of RNA from *Torpedo californica* experiments.

RNA totals

Electric organ SVs fish 1

	SV	pH10	DM	RNase	pHDR
SV starting material protein (mg)	.75	.75	.75	.75	.75
RNA recovered after treatment (ng)	8142	10092	8958	450	372
Amount of RNA loaded into gel (ng) **Fig 1c**	452	560	498	375	310
Average sample RNA (SV,pH10,DM) (ng)	9064				
After RNAse RNA (ng)	450				
Fraction RNA RNase resistant	0.05				

Electric organ SVs fish 2

	SV	pH10	DM	RNase	pHDR
SV starting material protein (mg)	.81	.81	.81	.81	.81
RNA recovered after treatment (ng)	4926	5598	5874	258	258
Average sample RNA (SV,pH10,DM) (ng)	5466				
After RNAse RNA (ng)	258				
Fraction RNA RNase resistant	0.05				

Electric organ SVs fish 3

	SV	pH10	DM	RNase	pHDR
SV starting material protein (mg)	.63	.63	.63	.63	.63
RNA recovered after treatment (ng)	10284	10716	9492	372	486
Amount of RNA loaded into gel (ng) **Fig 2b**	285	298	264	206	270
Average sample RNA (SV,pH10,DM) (ng)	10164				
After RNAse RNA (ng)	372				
Fraction RNA RNase resistant	0.04				

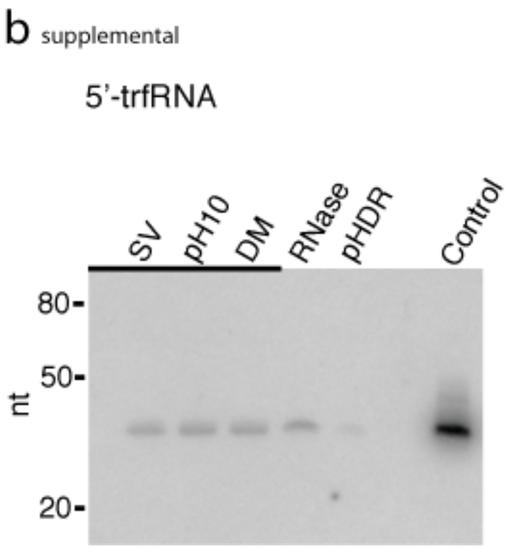

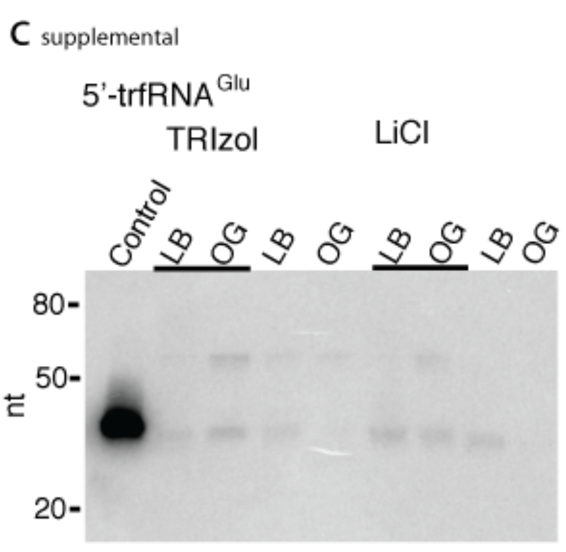

2b supplemental

	SV	pH10	DM	RNase	pHDR
Total RNA (ng) from preparation	10266	10716	9492	372	486
Amount of RNA (ng) loaded in Fig 2b lane	285	298	264	206	270

Northern Analysis - Calculation of 5'-trfRNA^{GLU}

Amount of trfRNA in Northern (ng)	13	17	17	14	3
Calculated amount of trfRNA from preparation (ng)	468	612	612	25	5
Average sample ttl trfRNA (SV,pH10,DM) (ng)	564				
After RNAse (ng)	25				
Fraction of 5'-trfRNA^{GLU} RNAse resistant	0.04				

2c supplemental – total RNA and LiCl sRNA enrichments from three *T. californica* rays.

0.1 gram of tissue used from each organ	Trizol		LiCl	
	Lobe (ng)	Organ (ng)	Lobe (ng)	Organ (ng)
Fish 1 Tissue	4872	1632	4987	4685
Fish 2 Tissue	4416	1957	5362	3343
Fish 3 Tissue	4459	1964	5388	3527
total	13747	5553	15737	11555

Calculations and legend continued on next page

	TRIzol				LiCl			
	LB	OG	LB	OG	LB	OG	LB	OG
Sample total and loaded RNA (ng) in Fig 2c lane	10998	4443	2749	1111	12590	9244	3147	2311

	TRIzol				LiCl			
Northern Analysis	LB	OG	LB	OG	LB	OG	LB	OG
Calculated amount of tRNA in Northern (ng)	0	4	0	0	0	2	0	0
Calculated amount of trfRNA in Northern (ng)	6	5	3	0	5	3	3	0

Supplemental Figure 2: b) Northern quantification based upon comparison to positive control and quantified using Imagequant. Calculations of 5'-trfRNA^{GLU} found in the SV preparation shown in Fig. 2b. c) Calculation of full-length tRNA$^{GLU}_{CUC}$ and 5'-trfRNA^{GLU} found in the electric lobe and organ of *T. californica* shown in Fig. 2c. TRIzol = total RNA preparation; LiCl = tRNA enriched preparation. First two lanes of LB and OG from each extraction method loaded with 80% sample (indicated by LB and OG); adjacent lanes of LB and OG loaded with 20% sample (4-fold dilution).

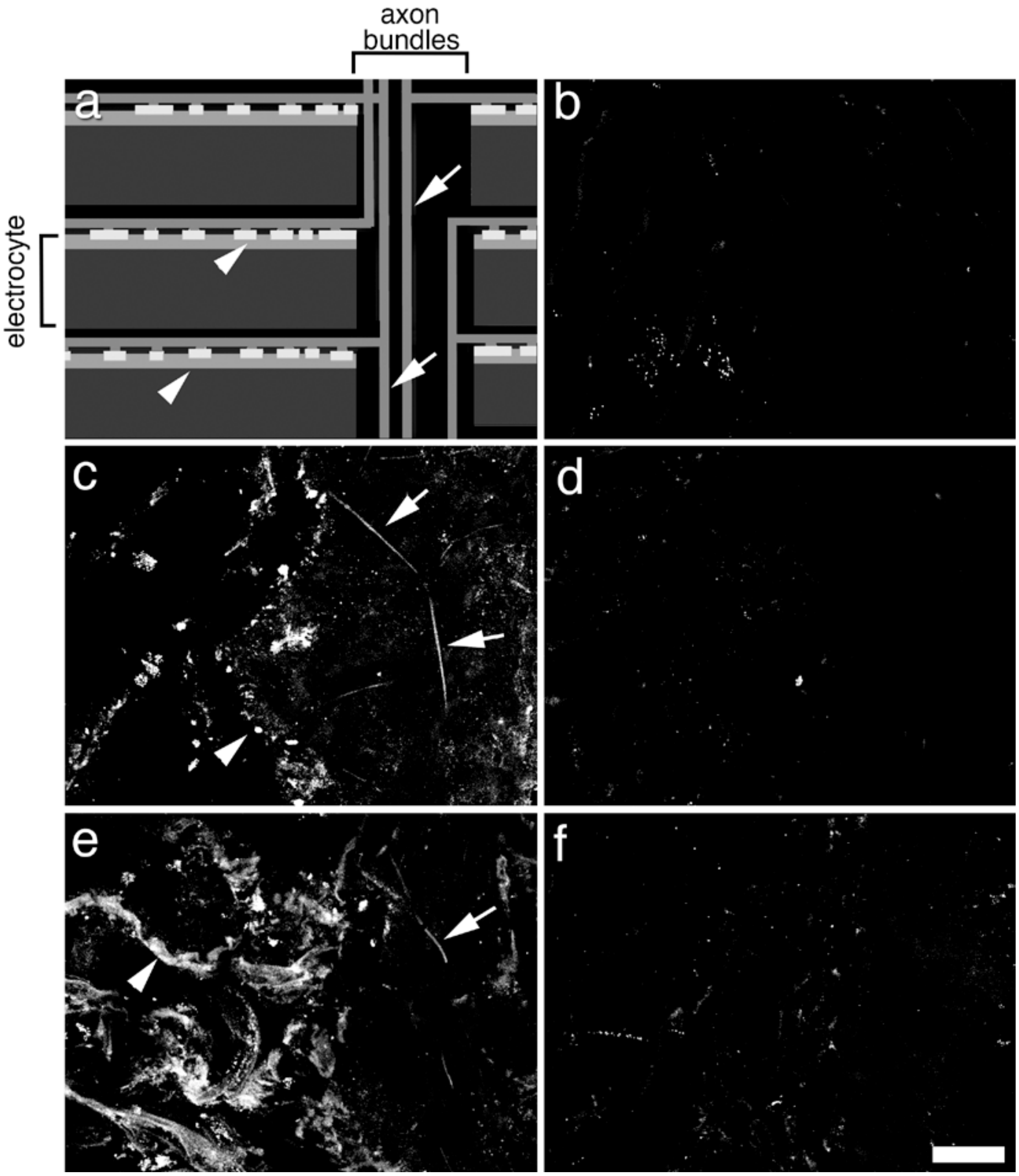

Supplemental Figure 3: In situ hybridization control demonstrating specificity of the *5'-trfRNA^{GLU}* *probe versus scrambled probe*. a) Cartoon depicting the orientation of cryostat sections of the electric organ shown in b-f. Stacks of electrocyte cells are on the left sides of each image, with the axon bundles and a small amount of an adjacent stack of electrocytes shown on the right side of each panel. Right side panels (b,d,f) show three *in situ* preparations labeled with the scrambled probe. No appreciable labeling can be found on tissue sections. Left panels c,e show two additional *in situ* preparations labeled with the probe for 5'-trfRNA^{GLU}. Axons, running nearly perpendicular to electrocyte cells are visible (arrowheads), and strong staining of electrocyte surfaces (arrowhead) can be seen. All images taken and balanced under identical conditions. Scale bar = 5 microns.

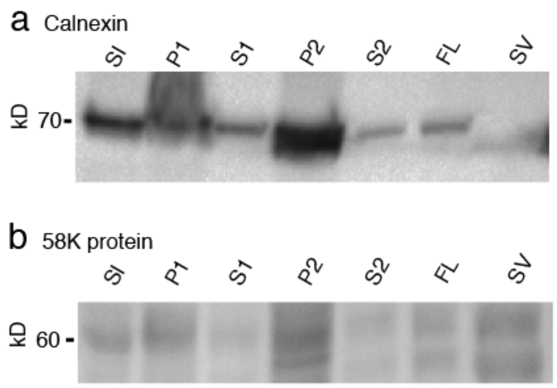

Supplemental Figure 4: Western-blot analysis of the synaptic vesicles during purification. The Endoplasmic reticulum protein Calnexin and the Golgi apparatus marker 58k protein were used as markers during the purification. The isolation procedure includes the collection of the original slurry (Sl), two centrifugation supernatants and pellets (S1, P1 and S2, P2), followed by a sucrose density gradient centrifugation and collection of the SV fluffy layer (FL). Further purification using size exclusion chromatography yields the final, enriched sample of SVs (SV). Vesicle preparation appears to contain little to no Calnexin or 58K protein. As a further control, SVs were isolated using an alternative, synaptosomal based strategy, and found to contain sRNAs of similar length (Supplemental Figure 6b).

Supplemental Table 2: Quantification of RNA from mouse brain SV experiments.

RNA totals

Mouse Brain preparation 1

	SV	pH10	DM	RNase	pHDR
SV starting material protein (mg)	.95	.95	.95	.95	.95
RNA recovered after treatment (ng)	17400	16662	15480	438	432
Amount of RNA loaded into gel (ng) **Fig 4c**	483	462	430	243	240
Average sample RNA (SV,pH10,DM) (ng)	16514				
After RNAse RNA (ng)	438				
Fraction RNA RNase resistant	0.03				

Mouse Brain preparation 2

	SV	pH10	DM	2 RNase	2 pHDR
SV starting material protein (mg)	.84	.84	.84	.84/ .84	.84/ .84
RNA recovered after treatment (ng)	9114	11910	11136	300/ 330	324/408
Amount of RNA loaded into gel (ng) **Fig 4d**	253	330	309	200	165
Amount of RNA loaded into gel (ng) **Fig 4e**	253	330	309	183	226
Average sample RNA (SV,pH10,DM) (ng)	10720				
After RNAse RNA (ng)	258				
Fraction RNA RNase resistant	0.03				

Mouse Brain preparation 3

	SV	pH10	DM	RNase	pHDR
SV starting material protein (mg)	.85	.85	.85	.85	.85
RNA recovered after treatment (ng)	19806	24702	22632	750	852
Average sample RNA (SV,pH10,DM) (ng)	22380				
After RNAse RNA (ng)	750				
Fraction RNA RNase resistant	0.03				

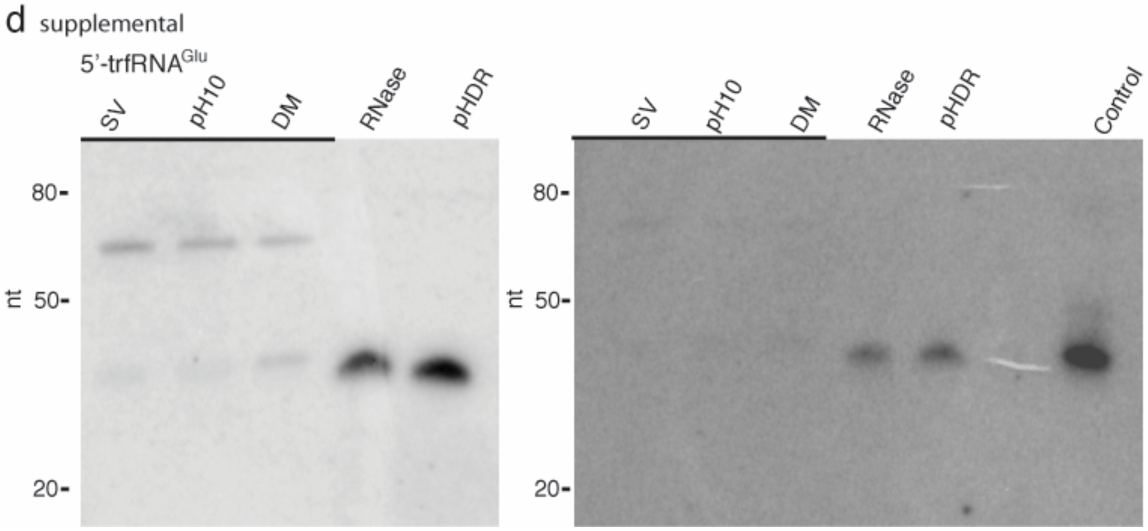

	SV	pH10	DM	RNase	pHDR
RNA recovered after treatment (ng)	9114	11910	11136	300	324
Amount of RNA loaded into gel (ng)	253	330	309	200	165

Northern Analysis

Amount of tRNA in northern (ng)	1.5	0.7	1	0	0
Calculated amount of tRNA after isolation (ng)	36	16.8	24	0	0
Amount of 5’-trfRNA^{GLU} in northern (ng)	1.2	2	2.5	10	14
Calculated amount of 5’-trfRNA^{GLU} after isolation (ng)	29	28	60	15	27
Amount of tRNA before RNAse	25.6				
Amount of 5’-trfRNA^{GLU} before RNAse	39				
Fraction of 5’-trfRNA^{GLU} RNAse resistant	0.25				

Amount of SVs (g)	8.4×10^{-4}
Number of Vesicles	3.28×10^{13}
Number of 5’-trfRNA^{Glu} (10ng)	3.73×10^{11}
Copies of 5’-trfRNA^{Glu} per vesicle	0.015

Supplemental Figure 5: Northern quantification based upon positive control of the 5’-trfRNA^{Glu} Control and quantified using ImageQuant. Left Figure – Used for publication; no control fragment was loaded in gel. Right Figure. Gel with similar loaded volume and control used as a basis for quantification. The number of synaptic vesicles can be estimated based upon the amount of vesicles used in the preparation (grams) and the published molecular weight of mouse CNS synaptic vesicles (25.6×10^{-18} g/vesicle)[1]. Likewise the amount of RNAse resistant copies of 5’-trfRNA^{Glu} per vesicle can be estimated based upon molecular mass of 5’-trfRNA^{Glu} and the amount found in the northern.

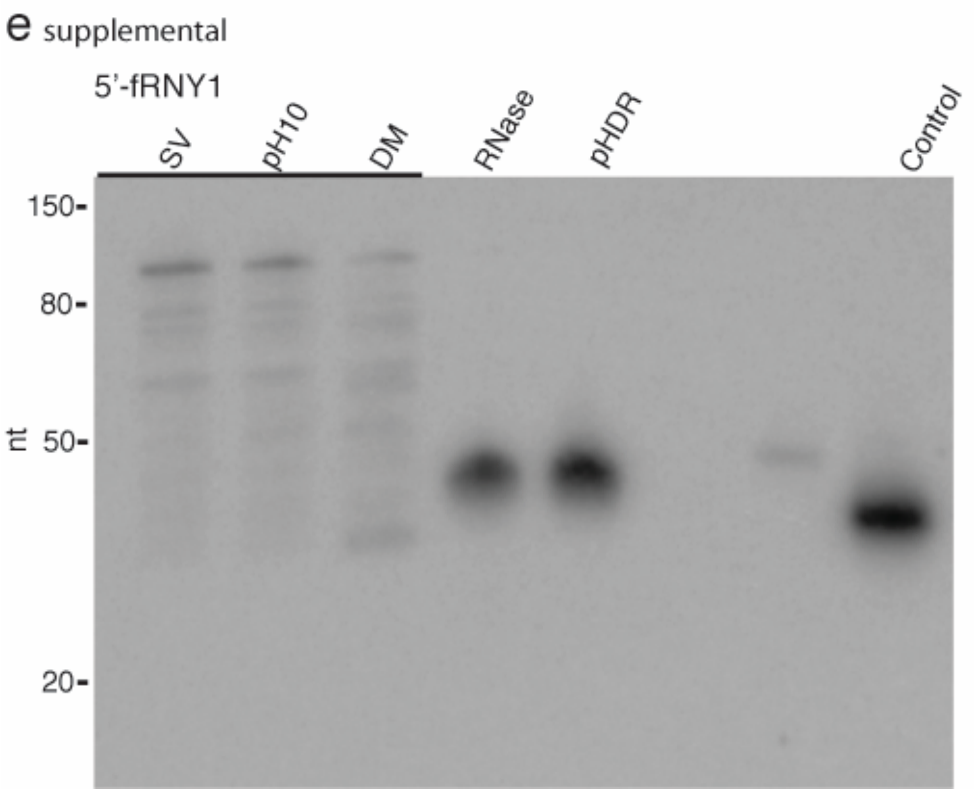

	SV	pH10	DM	RNase	pHDR
Sample total (ng)	9114	11910	11136	330	408
Sample loaded (ng)	253	330	309	183	226

Northern Analysis

Amount of 5'fRNY (ng) in northern	n/a	n/a	n/a	71	113
Calculated amount of 5'-fRNY after isolation (ng)	n/a	n/a	n/a	129	205

Amount of SVs (g) – see Sup Table 2	8.4×10^{-4}
Number of Vesicles	3.28×10^{13}
Number of 5'- fRNY1 (129ng)	4.65×10^{12}
Copies of 5'- fRNY1 per vesicle	0.142

Supplemental Figure 6: Northern quantification of 5'- fRNY1 in sample based upon positive control of the 5'-fRN Control loaded into the gel and quantified using ImageQuant. The number of synaptic vesicles can be estimated based upon the amount of vesicles used in the preparation (grams) and the published molecular weight of mouse CNS synaptic vesicles (25.6×10^{-18} g/vesicle)[1]. Likewise the amount of RNAse resistant copies of of 5'- fRNY1 per vesicle can be estimated based upon molecular mass of of 5'- fRNY1 and the amount found in the northern.

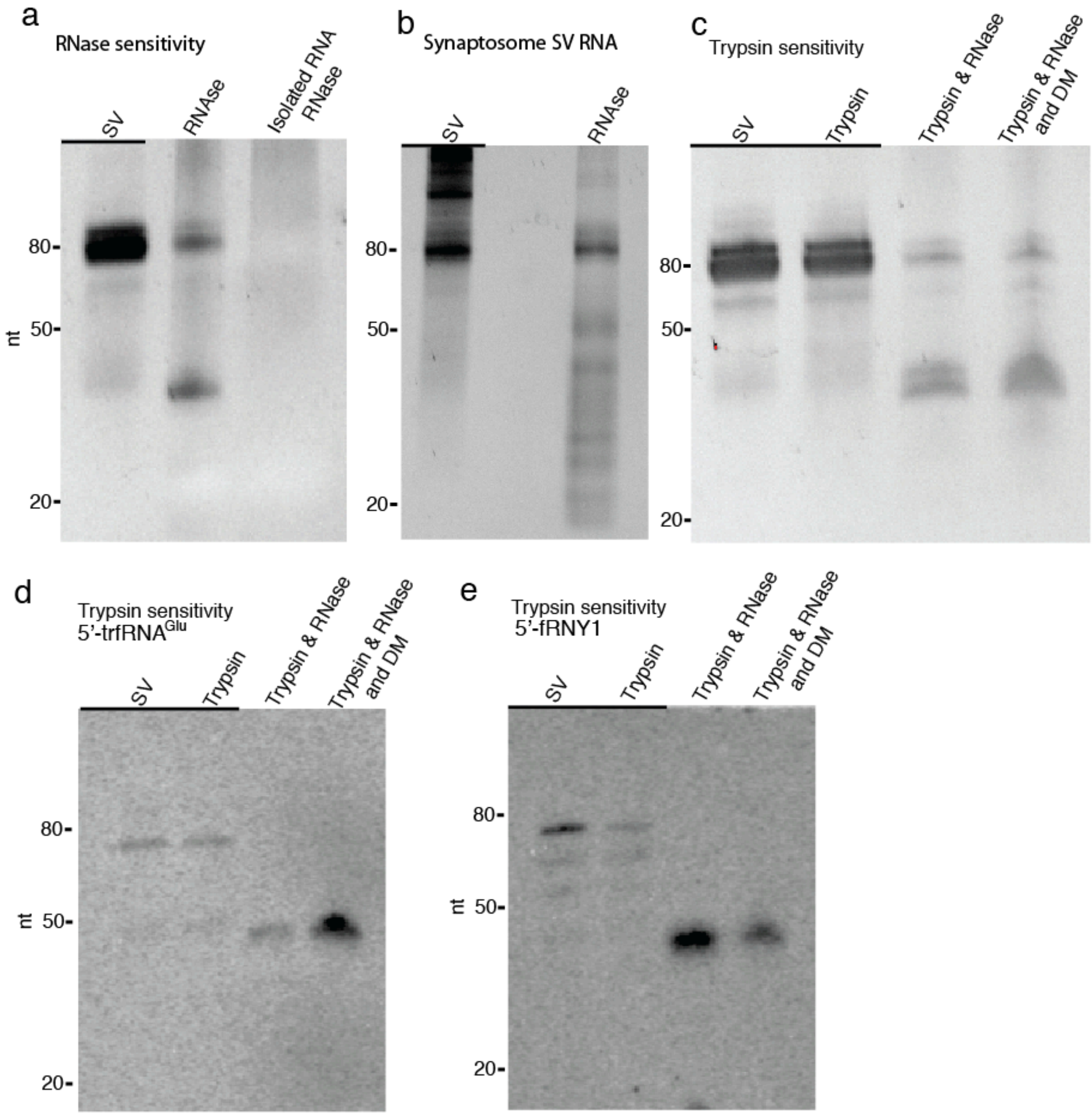

Supplemental Figure 7: RNase and Trypsin sensitivity, and alternative SV isolation procedure. a) Abundant sRNAs co-enrich with the synaptic vesicles (SV). After addition of RNase much of the RNA is degraded; however an RNase resistant ~32 nt band persists (RNAse). If the RNAse resistant RNA is isolated by TRIzol first, and then subjected to another round of RNase treatment, the RNA shows sensitivity to RNase and is degraded (Isolated RNA RNase). Bands of gel underlined (_), 20-fold reduction in loading b) SVs isolated by an alternative isolation procedure possess sRNAs. Vesicles were isolated following the synaptosomal isolation

procedure following Ahmed et al.[2] Isolated SVs were then treated with RNase as described - sRNAs of similar nt length can be seen (RNAse). c) sRNAs are resistant to trypsin treatment. SVs untreated shown in (SV), after trypsin treatment for 20 minutes (Trypsin), trypsin treatment for 20 minutes Trypsin followed by trypsin inhibitor and RNase (Trypsin and RNase), or trypsin treatment for 20 minutes in the presence of the membrane detergent DM, followed by trypsin inhibitor and RNase (Trypsin & RNase and DM). The RNase resistant sRNAs persisted - northerns for both (d) 5'-trfRNA^{Glu} and (e) 5'-fRNY1 demonstrate that the sRNAs were not affected by prior treatment with trypsin, and the addition of DM did not lead to degradation of either sRNA. The PBS perfused mouse brains used in (b) were kindly provided by Matthew Lee and Mendell Rimer under protocols approved by the IACUC of Texas A&M University.

References

1 Takamori, S. *et al.* Molecular anatomy of a trafficking organelle. *Cell* **127**, 831-846 (2006).

2 Ahmed, S., Holt, M., Riedel, D. & Jahn, R. Small-scale isolation of synaptic vesicles from mammalian brain. *Nature protocols* **8**, 998-1009, doi:10.1038/nprot.2013.053 (2013).
